# Novel heteroaryl phosphonicdiamides PTPs inhibitors as anti-hyperglycemic agents

**DOI:** 10.1186/s40199-014-0076-3

**Published:** 2014-12-27

**Authors:** Kuruva Chandra Sekhar, Rasheed Syed, Madhava Golla, Jyothi Kumar MV, Nanda Kumar Yellapu, Appa Rao Chippada, Naga Raju Chamarthi

**Affiliations:** Department of Chemistry, Sri Venkateswara University, Tirupati, 517 502 India; Department of Biotechnology, Sri Venkateswara University, Tirupati, 517 502 India; Biomedical informatics Center, Vector Control Research Centre, Indian Council of Medical Research, Puducherry, 605006 India; Department of Biochemistry, Sri Venkateswara University, Tirupati, 517 502 India

## Abstract

**Background:**

Chronic and oral administration of benzylamine improves glucose tolerance. Picolylamine is a selective functional antagonist of the human adenosine A_2B_ receptor. Phosphonic diamide derivatives enhance the cellular permeability and in turn their biological activities.

**Methods:**

A series of heteroaryl phosphonicdiamide derivatives were designed as therapeutics to control and manage type2 diabetes. Initially defined Lipinski parameters encouraged them as safer drugs. Molecular docking of these compounds against Protein tyrosine phosphatase (PTP), the potential therapeutic target of type 2 diabetes, revealed their potential binding ability explaining their anti-diabetic activity in terms of PTP inhibition. Human intestinal absorption, Caco-2 cell permeability, MDCK cell permeability, BBB penetration, skin permeability and plasma protein binding abilities of the title compounds were calculated by PreADMET server. A convenient method has been developed for the synthesis of title compounds through the formation of 1-ethoxy-N,N’-bis(4-fluorobenzyl/pyridin-3-ylmethyl)phosphinediamine by the reaction of 4-fluorobenzylamine/ 3-picolylamine with ethyldichlorophosphite, subsequently reacted with heteroaryl halides using lanthanum(III) chloride as a catalyst.

**Results:**

All the compounds exhibited significant *in vitro* anti-oxidant activity and *in vivo* evaluation in streptozotocin induced diabetic rat models revealed that the normal glycemic levels were observed on 12^th^ day by **9a** and 20^th^ day by **5b**, **5c**, **9e** and **9f.** The remaining compounds also exhibited normal glycemic levels by 25^th^ day.

**Conclusion:**

The results from molecular modeling, *in vitro* and *in vivo* studies are suggesting them as safer and effective therapeutic agents against type2 diabetes.

**Graphical Abstract:**
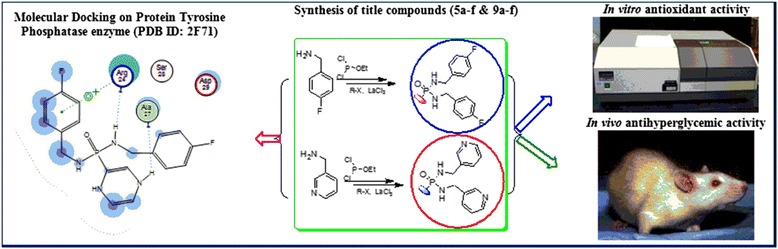
Development of PTPs inhibitors.

**Electronic supplementary material:**

The online version of this article (doi:10.1186/s40199-014-0076-3) contains supplementary material, which is available to authorized users.

## Background

**Figure 1:**
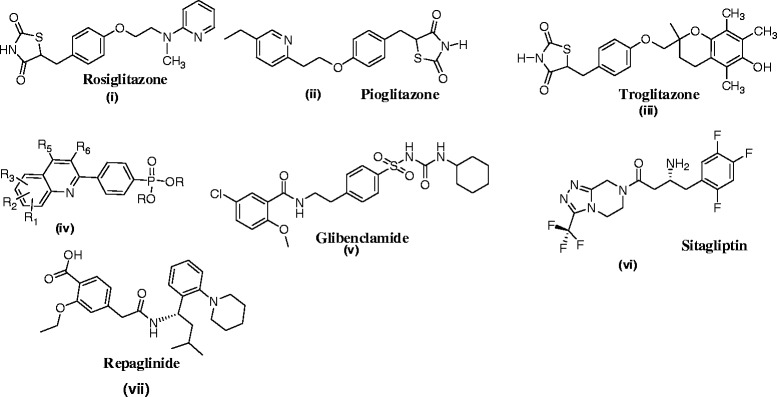
**A few anti-diabetic drugs.**

**Figure 2:**
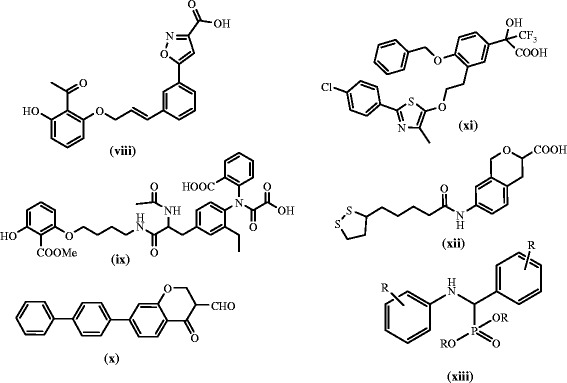
**Some of the PTP1B inhibitors Ref [** 6 **-** 11 **].**

The stipulation of anti-diabetic drugs is snowballing hastily, due to millions of people is distressing about diabetes. Several budding essential mechanisms for diabetes are characterized by elevation of blood glucose levels caused by decreased production of the hormone insulin and/or increased resistance to the action of insulin by certain cells. Tyrosine phosphorylation is associated with a group of enzymes which are mainly involved in the negative regulation of insulin signaling and intertwined in the insulin resistance, complementary to type 2 diabetes [1,2]. Protein tyrosine phosphatase-1B (PTP-1B) is one of the PTP enzymes a major negative regulator in both insulin and leptin signaling. It has been observed to serve as an outstanding target for the treatment of cancer, diabetes and obesity [3]. Mice lacking the PTP-1B have enhanced insulin sensitivity which certifies that the inhibition activity of PTP-1B could be a novel way of treating type 2 diabetes and obesity [1,2]. Thus insulin action will be enhanced by persuading the activity of cellular PTPases and glucose production can be reduced [4,5]. This study created an interest in designing the new drugs by structural modification of existing drugs (Figures 1 and 2).

The study of the reported drugs **i**-**vii** reveals that they are ideal for anti-diabetic activity due to the thiazolidine-2,4-dione **(i, ii, iii)**, pyridinyl (**i,ii**), quinolone (**iv),** urea and amide **(v, vii),** Flouro substituted, heteroaryl pyrazine **(vi)** and benzyl amine (**vii**). Compound **xiii** is a α-aminophosphonate with established anti-diabetic property which gave an idea to focus on phosphorus containing drugs.

Benzylamine is used to treat diabetes in traditional medicine. Chronic and oral administration of benzylamine improves glucose tolerance and the circulating lipid profile without increasing oxidative stress in overweight and pre-diabetic mice [12]. The stipulation of picolylamine was attested in the synthesis of various pharmacological compounds such as ^99m^Tc(I)-complexs [13] and selective functional antagonists of the human adenosine A_2B_ receptor [14]. When compared to normal benzyl amine analogues, picolylamine analogues are exhibiting the potential pharmacological activity [15]. Among the 2-picolyl, 3-picolyl and 4-picolyl amines, the performance of 3-picolyl amines are virtuous [16].

Phosphonic diamide derivatives enhance the cellular permeability and in turn their activities akin to the analogous phosphoric diamide prodrugs of 3′-azido-3′-deoxythymidine (AZT) monophosphate with AZT [17], glycine methyl ester phosphonic diamide of a 9-[2-(phosphonomethoxy)ethyl]-adenine (PMEA) analogue [18], and diamides of 9-[2-(phosphonomethoxy)ethyl]-N6-(cyclopropyl)-2-aminoadenine [19]. If phosphonic diamides hydrolyze *in vivo* to produce phosphonic acids benzyl amine itself act as antidiabetic agent [12]. Phosphonic diamide derivatives are used as prodrugs to improve the membrane permeability of drugs. P-C bond is playing an important role in preserving so many syndromes and in the synthesis of numerous anticancer [20], antiviral [21], antimicrobial [22], anti-diabetic [23], and antioxidant agents [24]. If the carbon in the P-C bond is aromatic, it acts better than the aliphatic carbon. Quinolines are expressed as LXR mediate disease inhibitors [25]. Quinoline phosphonicdiesters are known for preventing hypercholesterolemia and diabetes [26]. There are number of patents which are dependent on this type of drugs.

Lipinski parameters help in preclinical trials to avoid the tedious and costly procedures that can define them as drugs and to avoid the failure rates. Lipinski parameters suggest the potency of the compounds with a variety of molecular descriptors [27]. The *in silico* studies involving construction, optimization and molecular dynamics will generate the stable conformations of the molecules. It is also an important task to find out the structure based intermolecular interactions of the compounds with the biologically meaningful and effective targets at specified conditions [28]. This helps to predict the inhibitory activity and the strength of the molecule to form a stable complex with the target. The identification of binding orientations of the compounds in the binding site of target will provide fruitful information on their reactivity. Hence, in the present study we applied Lipinski parameters and molecular docking studies to predict the drug likeliness and binding ability of the compounds to the protein tyrosine phosphatase.

Although several synthetic methods are described for the preparation of such P-C bond containing compounds, one of them is the Michaelis–Arbuzov reaction. Unfortunately, it has some drawbacks when use classical conditions such as length of reaction time, high temperature and removal of the trialkyl phosphite used in a large excess. These drastic conditions may be responsible for side reactions, low yields and limits the application of such reactions to sensitive substrates. Recently, researchers focused on rare earth elemental catalysts due to their high catalytic properties and also act as Lewis acids. In this connection, we selected Lanthanum (III) chloride as an efficient catalyst for nucleophilic substitution on hetero aromatic ring for the synthesis of heteroaryl phosphonicdiamide derivatives *via* Michaelis-Arbuzov reaction.

The improved production and ineffective scavenging of reactive oxygen species (ROS) cause chemical changes in virtually all cellular components, leading to lipid peroxidation. The enhanced production of free radicals and oxidative stress is central event to the development of diabetic complications. This was supported by demonstration of increased levels of indicators of oxidative stress in diabetic individuals suffering from complications [29]. Oxidative stress is involved in the pathogenesis of diabetes and its complications. Use of antioxidants reduces oxidative stress and alleviates diabetic complications [30]. There are many reports on effects of antioxidants in the management of diabetes [31,32]. So the *in vitro* antioxidant activity was carried out as preliminary test for all the title compounds. The results of antioxidant activity supported for the reduction of oxidative stress. Finally, title compounds were screened for their *in vivo* anti-diabetic activity on mice. Most of the title compounds are effective and satisfactory in reducing glucose levels in both the tests.

## Materials and methods

### Chemistry

Chemicals were procured from Sigma–Aldrich and Merck were used as such without further purification. All solvents used for spectroscopic and other physical studies were reagent grade and were further purified by literature methods [33]. Melting points (m p) were determined by Guna Digital Melting Point apparatus using a calibrated thermometer. They expressed in degrees centigrade (°C) and are uncorrected. Infrared spectra (IR) were obtained on a Perkin-Elmer Model 281-B spectrophotometer. Samples were analyzed as potassium bromide (KBr) disks. Absorptions were reported in wave numbers (cm^−1^). ^1^H and ^13^C NMR spectra were recorded as solutions in DMSO-*d*_*6*_ on a Bruker AMX 400 MHz spectrometer operating at 400 MHz for ^1^H, 100 MHz for ^13^C and 161.9 MHz for ^31^P NMR. The ^1^H and ^13^C chemical shifts were expressed in parts per million (ppm) with reference to tetramethylsilane (TMS) and ^31^P chemical shifts to 85% H_3_PO_4_. LCMS mass spectra were recorded on a Jeol SX 102 DA/600 Mass spectrometer.

#### Synthesis of N,N’-di(4-fluorobenzyl)(2-pyrazinyl)phosphonic diamide (5a)

**Scheme 1:**
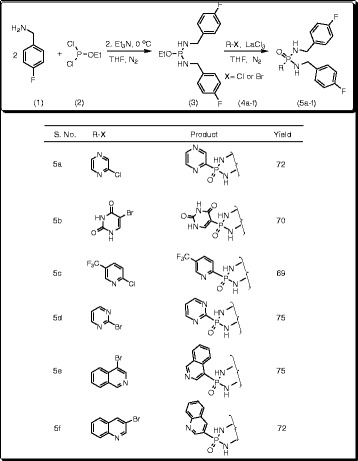
**Synthesis of substituted heteroaryl-N,N’-di(4-fluorobenzyl) phosphonicdiamides (5a-f).**

To a stirred solution of 4-fluorobenzylamine (0.002 mol) in dry tetrahydrofuran (THF) (10 mL), ethyldichlorophosphite (0.001 mol) was added at 0°C in the presence of triethylamine (TEA) (0.002 mol) under N_2_ atmosphere. After completion of the addition, the reaction mixture was heated to 30°C and stirred for 2 h to form the intermediate 1-ethoxy-N,N’-bis(4-fluorobenzyl)phosphinediamine (**3**). The reaction progress was monitored by thin layer chromatography (TLC) using ethyl acetate: hexane (1:1) as mobile phase. After completion of the reaction, it was filtered to remove triethylamine hydrochloride. 2-Chloropyrazine (**4a**) (0.001 mol) in dry THF (10 mL) was added to the filtrate under N_2_ atmosphere in the presence of La(III)Cl_3_.7H_2_O (20 mol%) and the reaction mixture was refluxed for 3 h. The progress of the reaction was monitored by TLC using ethyl acetate: hexane (1:1) as mobile phase. After completion of the reaction, catalyst was removed by filtration and the filtrate was concentrated in vacuum to afford the crude product. It was purified by silica gel column chromatography eluting with ethyl acetate: hexane (1:2) mixture to afford the title compound, *N*,*N*’-di(4-fluorobenzyl)(2-pyrazinyl)phosphonic diamide (**5a**). The same experimental procedure was adopted for the preparation of the remaining title compounds **5b-f** (Scheme 1).

### Spectral data

#### N,N’-Di(4-fluorobenzyl)(2-pyrazinyl)phosphonic diamide (5a)

Yield: 72%; mp: 162-164°C; IR (KBr): ῡ 3378 (N-H), 1252 (P = O), 1018 (P-C_Ar_) cm^−1^; ^1^H NMR (400 MHz, DMSO-*d*_*6*_): *δ* 8.52-6.84 (11H, m, Ar), 5.12 (2H, brs, H-8), 4.08-3.83 (4H, m, H-7); ^13^C NMR (100 MHz, DMSO-*d*_*6*_): *δ* 161.3 (C-4), 154.3 (C-1), 152.2 (C-1′), 148.9 (C-6′), 145.6 (C-3′), 147.9 (C-4′), 122.4-121.5 (C-2 & C-6), 117.4-116.2 (C-3 & C-5), 31.2 (C-7); ^31^P NMR (161.9 MHz, DMSO-d_*6*_): *δ* 28.9; LC MS (%): m/z 375.7 (100%) [MH^+•^]; Anal. Calcd. for C_18_H_17_N_4_F_2_OP: C 57.76; H 4.58; N 14.97; Found: C 57.63; H 4.39; N 14.77.

#### 2,4-Dioxo-1,2,3,4-tetrahydro-5-pyrimidinyl-N,N’-di(4-fluorobenzyl)phosphonic diamide (5b)

Yield: 70%; mp: 189-191°C; IR (KBr): ῡ 3386 (N-H), 1238 (P = O), 992 (P-C_Ar_) cm^−1^; ^1^H NMR (400 MHz, DMSO-*d*_*6*_): *δ* 9.06 (1H, brs, H-3′), 8.32-6.74 (9H, m, Ar), 5.73-5.68 (1H, s, H-5′), 5.15 (2H, brs, H-8), 3.83-4.08 (4H, m, H-7); ^13^C NMR (100 MHz, DMSO-*d*_*6*_): *δ* 169.6 (C-2′), 161.6 (C-4), 161.3 (C-4′), 158.2 (C-1′), 155.4 (C-1), 141.9 (C-6′), 122.6-121.8 (C-2 & C-6), 117.8-116.8 (C-3 &C-5), 30.9 (C-7); ^31^P NMR (161.9 MHz, DMSO-d_*6*_): *δ* 27.6; LC MS (%): m/z 407.8 (100%) [MH^+•^]; Anal. Calcd. for C_18_H_17_N_4_F_2_O_3_P: C 53.21; H 4.22; N 13.79; Found: C 53.08; H 4.12; N 13.55.

#### N,N’-Di(4-fluorobenzyl)[5-(trifluoromethyl)-2-pyridyl]phosphonic diamide (5c)

Yield: 69%; mp: 202-204°C; IR (KBr): ῡ 3354 (N-H), 1261 (P = O), 1010 (P-C_Ar_) cm^−1^; ^1^H NMR (400 MHz, DMSO-*d*_*6*_): *δ* 7.65-6.50 (11H, m, Ar), 5.12 (2H, brs, H-8), 4.06-3.83 (4H, m, H-7); ^13^C NMR (100 MHz, DMSO-*d*_*6*_): *δ* 159.1 (C-4), 155.9 (C-1′), 154.8 (C-3′), 154.0 (C-1), 133.6 (C-5′), 133.5 (C-4′), 122.2-121.1 (C-2 & C-6), 119.6 (C-6′), 118.9 (C-7′), 117.7-116.6 (C-3 & C-5), 30.9 (C-7); ^31^P NMR (161.9 MHz, DMSO-d_*6*_): *δ* 28.7; LC MS (%): m/z 442.8 (100%) [MH^+•^]; Anal. Calcd. for C_20_H_17_F_5_N_3_OP: C 54.43; H 3.88; N 9.52; Found: 54.15; H 3.51; N 9.22.

#### N,N’-Di(4-fluorobenzyl)(2-pyrimidinyl)phosphonic diamide (5d)

Yield: 75%; mp: 167-169°C; IR (KBr): ῡ 3346 (N-H), 1268 (P = O), 996 (P-C_Ar_) cm^−1^; ^1^H NMR (400 MHz, DMSO-*d*_*6*_): *δ* 8.55-6.79 (11H, m, Ar), 5.14 (2H, brs, H-8), 4.08-3.83 (4H, m, H-7); ^13^C NMR (100 MHz, DMSO-*d*_*6*_): *δ* 162.5 (C-1′), 161.9 (C-4), 155.1 (C-1), 154.3 (C-3′ & C-5′), 124.4 (C-4′), 121.8-121.1 (C-2 & C-6), 117.6-116.4 (C-3 & C-5), 31.4 (C-7); ^31^P NMR (161.9 MHz, DMSO-d_*6*_): *δ* 28.2; LC MS (%): m/z 375.4 (100%) [MH^+•^]; Anal. Calcd. for C_18_H_17_F_2_N_4_OP: C 57.76; H 4.58; N 14.97; Found: 57.62; H 4.41; N 14.82.

#### N,N’-Di(4-fluorobenzyl)(4-isoquinolyl)phosphonic diamide (5e)

Yield: 75%; mp: 221-224°C; IR (KBr): 3364 (N-H), 1274 (P = O), 986 (P-C_Ar_) cm^−1^; ^1^H NMR (400 MHz, DMSO-*d*_*6*_): *δ* 8.65-6.76 (14H, m, Ar), 5.16 (2H, brs, H-8), 4.06-3.84 (4H, m, H-7); ^13^C NMR (100 MHz, DMSO-*d*_*6*_): *δ* 161.6 (C-4), 156.2 (C-4′), 155.2 (C-1), 142.6 (C-2′), 135.9 (C-9′), 129.5 (C-7′), 129.2 (C-10′), 127.5 (C-5′), 127.1 (C-8′), 126.9 (C-6′), 126.2 (C-1′), 122.3-121.5 (C-2 & C-6), 117.7-116.6 (C-3 & C-5), 30.7 (C-7); ^31^P NMR (161.9 MHz, DMSO-d_*6*_): *δ* 27.8; LC MS (%): m/z 424.5 (100%) [MH^+•^]; Anal. Calcd. for C_23_H_20_F_2_N_3_OP: C 65.25; H 4.76; N 9.92; Found: C 65.09; H 4.57; N 9.71.

#### N,N’-Di(4-fluorobenzyl)(3-quinolyl)phosphonic diamide (5f)

Yield: 72%; mp: 179-181°C; IR (KBr): 3372 (N-H), 1259 (P = O), 1012 (P-C_Ar_) cm^−1^; ^1^H NMR (400 MHz, DMSO-*d*_*6*_): *δ* 8.63-6.92 (14H, m, Ar), 5.13 (2H, brs, H-8), 4.08-3.83 (4H, m, H-7); ^13^C NMR (100 MHz, DMSO-*d*_*6*_): *δ* 161.5 (C-4), 157.2 (C-9′), 154.5 (C-1), 148.4 (C-2′), 136.8 (C-3′), 131.7 (C-6′), 128.5 (C-4′), 127.3 (C-7′), 126.8 (C-10′), 126.7 (C-5′), 123.6 (C-8′), 122.1-121.3 (C-2 & C-6), 117.8-116.2 (C-3 & C-5), 30.5 (C-7); ^31^P NMR (161.9 MHz, DMSO-d_*6*_): *δ* 28.9; LC MS (%): m/z 424.6 (100%) [MH^+•^]; Anal. Calcd. for C_23_H_20_F_2_N_3_OP: C 65.25; H 4.76; N 9.92; Found: C 65.08; H 4.51; N 9.84.

#### Synthesis of 4,6-dimethoxy-1,3,5-triazin-2-yl-N,N’-di(3-pyridylmethyl)phosphonicdiamide (9a)

**Scheme 2:**
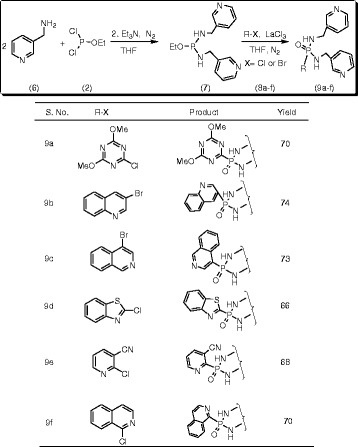
**Synthesis of substituted heteroaryl-N,N’-di(3-pyridylmethyl) phosphonicdiamides (9a-f).**

To a stirred solution of 3-picolylamine (**6**) (0. 002 mol) in dry tetrahydrofuran (THF) (10 mL), ethyldichlorophosphite (**2**) (0.001 mol) was added at 0°C in the presence of triethylamine (TEA) (0.002 mol) under N_2_ atmosphere. After completion of the addition, the reaction mixture was raised to 30°C and stirred for 2 h to form the intermediate 1-ethoxy-N,N’-bis(pyridin-3-ylmethyl)phosphinediamine (**7**). The reaction progress was monitored by thin layer chromatography (TLC) using ethyl acetate: hexane (1:1) as mobile phase. After completion of the reaction, it was filtered to remove triethylamine hydrochloride. 2-Chloro-4,6-dimethoxy-1,3,5-triazine (**8a**) (0.001 mol) in dry THF (10 mL) was added to the filtrate under N_2_ atmosphere at 20°C in the presence of La(III)Cl_3_.7H_2_O (20 mol%) and the reaction mixture was refluxed for 3 h. The progress of the reaction was monitored by TLC using ethyl acetate: hexane (1:1). After completion of the reaction, catalyst was removed by filtration and the filtrate was concentrated in rota-evaporator to afford the crude product. It was purified by silica gel column chromatography eluting with ethyl acetate: hexane (1:2) mixture to afford the title compound, 4,6-dimethoxy-1,3,5-triazin-2-yl-*N*,*N*’-di(3-pyridylmethyl)phosphonic diamide (**9a**). The same experimental procedure was adopted for the preparation of the remaining title compounds **9b-f** (Scheme 2).

### Spectral data

#### 4,6-Dimethoxy-1,3,5-triazin-2-yl-N,N’-di(3-pyridylmethyl)phosphonic diamide (9a)

Yield: 70%; mp: 198-200°C; IR (KBr): ῡ 3371 (N-H), 1242 (P = O), 989 (P-C_Ar_) cm^−1^; ^1^H NMR (400 MHz, DMSO-*d*_*6*_): *δ* 8.92-7.26 (8H, m, Ar), 5.63 (2H, brs, H-8), 4.39-4.35 (4H, d, H-7), 3.72 (6H, s, -OMe); ^13^C NMR (100 MHz, DMSO-*d*_*6*_): *δ* 178.3 (C-3′ & C-5′), 169.2 (C-1′), 149.5 (C-2), 148.1 (C-4), 145.8 (C-1), 135.3 (C-6), 121.5 (C-5), 51.9 (C-OMe), 38.6 (C-7); ^31^P NMR (161.9 MHz, DMSO-d_*6*_): *δ* 21.6; LC MS (%): m/z 402.5 (100%) [MH^+•^]; Anal. Calcd. for C_17_H_20_N_7_O_3_P: C 50.87; H 5.02; N 24.43; Found: C 50.66; H 4.81; N 24.23.

#### N,N’-Di(3-pyridylmethyl)(3-quinolyl)phosphonic diamide (9b)

Yield: 74%; mp: 206-208°C; IR (KBr): 3376 (N-H), 1247 (P = O), 1005 (P-C_Ar_) cm^−1^; ^1^H NMR (400 MHz, DMSO-*d*_*6*_): *δ* 8.61-7.46 (14H, m, Ar), 5.64 (2H, brs, H-8), 4.35-4.31 (4H, d, H-7); ^13^C NMR (100 MHz, DMSO-*d*_*6*_): *δ* 149.4 (C-2), 148.9 (C-4), 147.5 (C-2′), 145.2 (C-9′), 135.3 (C-6), 134.6 (C-1), 132.3 (C-3′), 131.7 (C-6′), 128.9 (C-4′), 127.5 (C-5′), 127.3 (C-10′), 126.1 (C-7′), 122.5 (C-8′), 121.7 (C-5), 38.8 (C-7); ^31^P NMR (161.9 MHz, DMSO-d_*6*_): *δ* 20.2; LC MS (%): m/z 390.3 (100%) [MH^+•^]; Anal. Calcd. for C_21_H_20_N_5_OP: C 64.77; H 5.18; N 17.99; Found: C 64.51; H 5.03; N 17.81.

#### 4-Isoquinolyl-N,N’-di(3-pyridylmethyl)phosphonic diamide (9c)

Yield: 73%; mp: 166-169°C; IR (KBr): ῡ 3379 (N-H), 1253 (P = O), 995 (P-C_Ar_) cm^−1^; ^1^H NMR (400 MHz, DMSO-*d*_*6*_): *δ* 8.63-7.45 (14H, m, Ar), 5.62 (2H, brs, H-8), 4.37-4.34 (4H, d, H-7); ^13^C NMR (100 MHz, DMSO-*d*_*6*_): *δ* 152.5 (C-4′), 149.1 (C-2), 148.5 (C-4), 145.2 (C-1), 143.9 (C-2′), 135.1 (C-6), 134.5 (C-9′), 130.2 (C-10′), 129.5 (C-7′), 128.2 (C-6′), 127.4 (C-5′), 127.1 (C-8′), 125.9 (C-1′), 121.3 (C-5), 38.4 (C-7); ^31^P NMR (161.9 MHz, DMSO-d_*6*_): *δ* 19.1; LC MS (%): m/z 390.5 (100%) [MH^+•^]; Anal. Calcd. for C_21_H_20_N_5_OP: C 64.77; H 5.18; N 17.99; Found: C 64.68; H 5.12; N 17.85.

#### 1,3-Benzothiazol-2-yl-N,N’-di(3-pyridylmethyl)phosphonic diamide (9d)

Yield: 66%; mp: 175-177°C; IR (KBr): ῡ 3383 (N-H), 1245 (P = O), 1013 (P-C_Ar_) cm^−1^; ^1^H NMR (400 MHz, DMSO-*d*_*6*_): *δ* 8.48-7.44 (12H, m, Ar), 5.62 (2H, brs, H-8), 4.35-4.31 (4H, d, H-7); ^13^C NMR (100 MHz, DMSO-*d*_*6*_): *δ* 162.2 (C-1′), 155.3 (C-8′), 149.2 (C-2), 148.9 (C-4), 145.5 (C-1), 135.8 (C-6), 134.3 (C-9′), 129.5 (C-4′), 127.1 (C-5′), 125.9 (C-6′), 125.7 (C-3′), 121.2 (C-5), 38.3 (C-7); ^31^P NMR (161.9 MHz, DMSO-d_*6*_): *δ* 21.6; LC MS (%): m/z 396.5 (100%) [MH^+•^]; Anal. Calcd. for C_19_H_18_N_5_OPS: C 57.71; H 4.59; N 17.71; Found: C 57.62; H 4.41; N 17.58.

#### 3-Cyano-2-pyridyl-N,N’-di(3-pyridylmethyl)phosphonic diamide (9e)

Yield: 68%; mp: 172-174°C; IR (KBr): ῡ 3388 (N-H), 1258 (P = O), 1018 (P-C_Ar_) cm^−1^; ^1^H NMR (400 MHz, DMSO-*d*_*6*_): *δ* 8.66-7.41 (11H, m, Ar), 5.63 (2H, brs, H-8), 4.32-4.30 (4H, d, H-7); ^13^C NMR (100 MHz, DMSO-*d*_*6*_): *δ* 157.3 (C-1′), 156.3 (C-3′), 149.3 (C-2), 148.8 (C-4), 145.5 (C-1), 138.4 (C-5′), 135.9 (C-4′), 135.8 (C-6), 121.4 (C-5), 118.3 (C-7′), 113.6 (C-6′), 38.2 (C-7); ^31^P NMR (161.9 MHz, DMSO-d_*6*_): *δ* 22.7; LC MS (%): m/z 365.7 (100%) [MH^+•^]; Anal. Calcd. for C_18_H_17_N_6_OP: C 59.34; H 4.70; N 23.07; Found: C 59.19; H 4.48; N 22.91.

#### 1-Isoquinolyl-N,N’-di(3-pyridylmethyl)phosphonic diamide (9f)

Yield: 73%; mp: 185-187°C; IR (KBr): ῡ 3387 (N-H), 1261 (P = O), 1010 (P-C_Ar_) cm^−1^; ^1^H NMR (400 MHz, DMSO-*d*_*6*_): *δ* 8.61-7.48 (14H, m, Ar), 5.64 (2H, brs, H-8), 4.34-4.31 (4H, d, H-7); ^13^C NMR (100 MHz, DMSO-*d*_*6*_): *δ* 159.3 (C-1′), 149.1 (C-2), 148.5 (C-4), 145.9 (C-1), 144.8 (C-3′), 135.9 (C-10′), 135.3 (C-6), 129.7 (C-6′), 129.5 (C-9′), 129.1 (C-8′), 128.5 (C-7′), 127.3 (C-5′), 122.6 (C-4′), 121.7 (C-5), 38.4 (C-7); ^31^P NMR (161.9 MHz, DMSO-d_*6*_): *δ* 18.9; LC MS (%): m/z 390.2 (100%) [MH^+•^]; Anal. Calcd. for C_21_H_20_N_5_OP: C 64.77; H 5.18; N 17.99; Found: C 64.55; H 5.02; N 17.75.

### Molecular modeling

All the *in silico* studies were carried out in the Molecular Operating Environment (MOE) software tool [34].

#### Protein preparation and processing

The three dimensional X-Ray Crystallographic structure of Protein tyrosine phosphatase (PTP) was retrieved from Protein Data Bank (PDB ID: 2F71). The structure was loaded into the MOE working environment ignoring the water molecules and hetero atoms. Polar hydrogens were added to the protein and subjected protonation followed by energy minimization in the implicit solvated environment in MMFF94x force field at a gradient cut off value of 0.05. A stabilized conformation of the protein was obtained after energy minimization and it was used for docking study.

#### Molecular docking

The above obtained stable conformation of the protein was preceded with molecular docking process. The binding site was defined with Arg 24, Asp 181, Ser 216, Ala 217, Gly 220, Arg 221 and Arg 254 residues. These are all the residues that were found to be interacting with the previously reported sulfamic acid inhibitor and hence considered for the docking of library of the present novel compounds. All the ligands were docked into the specified binding site using alpha triangle placement methodology where the Poses are generated by superposition of ligand atom triplets and triplets of receptor site points. A random triplet of ligand atoms and a random triplet of alpha sphere centers are used to determine the binding pose at each interaction. The free energy of binding of each compound from each pose generated after docking process is determined by London dG scoring function. A total of 30 conformations were generated for each compound and they were refined and rescored again using the same scoring function. The pose with lowest binding score was selected for further analysis and to analyze the binding mode orientations of the ligands in the binding site.

### ADMET study

Pharmacokinetic parameters like absorption, distribution, metabolism and excretion of compounds designates their disposition. Such parameters influence the pharmacokinetics of the drug in the body and in turn influence their performance and pharmacological activity [35]. In that sequence we have predicted some ADMET properties for the designed compounds to define them as drug candidates at their significant conditions. The parameters such as Caco-2 (colon adeno carcinoma) cell permeability, MDCK (Madin-Darby canine kidney) cell permeability, BBB (blood-brain barrier) penetration, HIA (human intestinal absorption), skin permeability and plasma protein binding ability were predicted by submitting the structures to PreADMET online software tool (http://preadmet.bmdrc.org/index.php?option=com_content&view=frontpage&Itemid=1) a web-based application server for predicting ADMET.

### Pharmacology

Compounds **5a-f** and **9a-f** were screened for *in vitro* antioxidant activity by DPPH (2,2-diphenyl-1-picrylhydrazyl), NO and H_2_O_2_ methods where Ascorbic acid and BHT (Butylated hydroxytoluene) as standards. Subsequently all the title compounds were screened for their *in vivo* antihyperglycemic activity in twenty five days period and examined for every four days. The experimental procedures are described below.

### Antioxidant activity

#### DPPH radical scavenging activity

The DPPH radical scavenging activity was measured from the bleaching of the purple colored methanol solution of 2,2-diphenyl-1-picrylhydrazyl (DPPH). Initially 1 mL of various concentrations of test compounds (50, 75, 100 and 150 μg/ mL) in methanol were added to 4 mL of 0.004% (w/v) methanol solution of DPPH. The resultant test solutions were incubated for 30 min period at room temperature and absorbance was read against blank at 517 nm. All the tests were carried out in triplicate. The % of inhibition (I%) of free radical production from DPPH was calculated by following equation.

$$ \mathrm{I}\% = \left[\left({\mathrm{A}}_{\mathrm{control}}\hbox{-} {\mathrm{A}}_{\mathrm{sample}}\right)/{\mathrm{A}}_{\mathrm{control}}\right]\mathrm{X}100 $$

#### Nitric oxide (NO) scavenging activity

NO scavenging activity action was measured by slightly modified method of Green et al. and Marcocci et al. [36]. The mixture of 1 mL of sodium nitro prusside (10 mM) and 1.5 mL of phosphate buffer saline (0.2 M, pH 7.4) were tested to different concentrations (50, 75, 100 and 150 μg/mL) of the test compounds and incubated for 150 min at 25°C and treated with 1 mL of Griess reagent and absorbance of the chromophore was measured at 546 nm. Butylated hydroxyl toluene was used as the standard in the present method. Tests were carried out in triplicate. Nitric oxide scavenging activity was calculated by the following equation.

$$ \%\ \mathrm{of}\ \mathrm{scavenging}=\left[\left({\mathrm{A}}_{\mathrm{control}}\hbox{-} {\mathrm{A}}_{\mathrm{sample}}\right)/{\mathrm{A}}_{\mathrm{control}}\right]\mathrm{X}100 $$

#### Hydrogen peroxide (H_2_O_2_) scavenging activity

Radical scavenging activity of the title compounds was screened against H_2_O_2_ through the method of Ruch et al. [37]. A solution of H_2_O_2_ (40 mM) in phosphate buffer (P^H^ 7.4) was prepared, 0.6 mL of prepared H_2_O_2_ solution was added to the test compounds at different concentrations (50, 75, 100 and 150 μg/mL) and the absorbance value for the reaction mixture was recorded at 230 nm for every test sample in average of triplicate. Tests were carried out in triplicate. The per cent of scavenging of H_2_O_2_ was calculated by the following equation.

$$ \%\ \mathrm{of}\ \mathrm{scavenging}=\left[\left({\mathrm{A}}_{\mathrm{control}}\hbox{-} {\mathrm{A}}_{\mathrm{sample}}\right)/{\mathrm{A}}_{\mathrm{control}}\right]\mathrm{X}100 $$

Where A_control_ is the absorbance of the control reaction (containing all reagents except the test compound) and A_sample_ is the absorbance of the test compound and Acetate buffer as A_blank_.

### *In vivo* antihyperglycemic activity

#### Induction of diabetes

Male wistar albino rats (body weight 180-200 grams) were subjected to intra-peritoneal administration of Streptozotocin dissolved in freshly prepared 0.01M ice-cold citrate buffer (P^H^ 4.3) at a dose of 50 mg/Kg body weight. After 72 hours, the animals with fasting blood glucose levels ≥350 mg/dL were used to evaluate the anti-diabetic activity of title compounds. Blood glucose levels were measured with the help of Accuchec Glucometer (Glucose oxidase method). All the animals were maintained in ventilated cages provided with standard pellet diet and water in light/dark cycle of (12h/12h) [38]. All of animal experiments were carried out according to the guidelines of the Sri Venkateswara University’s Institutional Animal Care and Use Committee (No./02(i)/a/CPCSCA/IAEC/SVU/TV).

#### Experimental design

The animals were divided into fifteen groups and each group maintained six rats. Group 1 as normal rats Untreated, Group 2 as diabetic rats Untreated, Group 3 as Diabetic rats treated with standard Glibenclamide (25 mg/kg b.w.) and Group 4-15 as Diabetic rats treated with title compounds (25 mg/kg b.w.) from **5a-f** and **9a-f** respectively for each group. After an overnight fast, the drug dissolved in DMSO (25 mg/kg b.w.) was fed to 4-15 group rats by gastric intubation using force feeding needle. Normal untreated and diabetic untreated rats were fed with normal diet and distilled water alone. Group 3 diabetic rats were treated with Glibenclamide 25 mg/kg b.w. Blood samples were collected to measure blood glucose levels from the tail vein on 1^st^, 4^th^, 8^th^, 12^th^, 16^th^, 20^th^ and 25^th^ days after the administration of drug and blood glucose levels were determined by glucose oxidase–peroxidase method [39].

## Results

### Chemistry

The IR spectra of **5a-f** showed the expected absorption bands at 998-1008, 3350–3330 and 1255–1233 cm^−1^ for the P-C_(Ar)_, NH and P = O stretching vibrations respectively [40]. The signals in *δ* 5.12-5.16 of **5a-f** and *δ* 5.63-5.64 of **9a-f** are representing the NH protons attached to the phosphorus atom. All ^13^C signal of aromatic carbon attached to the phosphorus is observed in between the range of *δ* 128-139 and *δ* 152-169. ^31^P NMR signals appeared in the range of 27.3 to 28.6 ppm as expected for the P = O group of the title compounds.

### Prediction of Lipinski parameters

The three dimensional structures were constructed for all the compounds and their stable conformations were obtained after optimization. These conformations were used to study their Lipinski parameters and the results showed that all of them are showing best properties with good agreement to Lipinski rule suggesting them as safer drugs. All the compounds have the molecular weight less than 500 Da, the lowest molecular weight of 364 Da was found with **9e** and the highest molecular weight of 441 Da was found with **5c**. The number of hydrogen bond donors is found to be less than 5 and the hydrogen bond acceptors is less than 10 for all the compounds. The logP values observed below 5 are themselves indicating that they are all non-toxic to the host system. The molar refractivity is also found to be in the optimal range of 40-150. The remaining descriptors like surface area, volume, hydration energy, polarizability and energy levels are also encouraging them with suitable features to bind and inhibit the target, there by better results can be expected and promotes them as safer and effective drugs (Table 1).

**Table 1:** **Lipinski parameters of the title compounds 5a-f and 9a-f**

**Ligand**	**Molecular Weight (Daltans)**	**Hydrogen Bond Donors**	**Hydrogen Bond Acceptors**	**LogP**	**Molar Refractivity (A** ^**o3**^ **)**	**Surface area (A** ^**o2**^ **)**	**Volume (A** ^**o3**^ **)**	**Hydration energy (K.cal/mol)**	**Polarizability (A** ^**o3**^ **)**	**Gradient energy (K.cal/molA** ^**o**^ **)**	**Total energy (K.cal/mol)**
**5a**	374	2	5	4.5	103.11	672.40	1175.38	−4.06	37.40	0.086231	157.989
**5b**	406	4	5	2.6	106.25	691.18	1199.19	−14.33	39.78	0.092319	142.533
**5c**	441	2	7	3.32	110.60	653.20	1247.31	−2.86	40.55	0.087467	168.395
**5d**	374	2	5	2.31	103.41	685.33	1177.04	−4.18	38.50	0.082136	158.398
**5e**	423	2	4	3.50	123.12	732.44	1318.93	−2.41	45.55	0.094976	196.071
**5f**	423	2	4	3.58	124.45	748.81	1328.20	−3.87	45.55	0.097266	190.509
**9a**	401	2	8	1.8	102.18	715.05	1257.28	−17.30	42.18	0.099135	166.626
**9b**	389	2	4	4.3	112.33	697.94	1253.43	−10.04	44.77	0.091709	166.626
**9c**	389	2	4	4.2	111.00	696.31	1259.79	−9.42	44.77	0.095766	188.333
**9d**	395	2	4	4.3	109.77	704.82	1243.68	−8.45	44.10	0.091985	170.187
**9e**	364	2	4	2.9	98.20	685.29	1201.59	−11.42	41.39	0.096933	245.03
**9f**	389	2	4	4.2	111.00	702.04	1267.84	−8.75	44.77	0.090542	186.537

### Molecular docking

**Figure 3:**
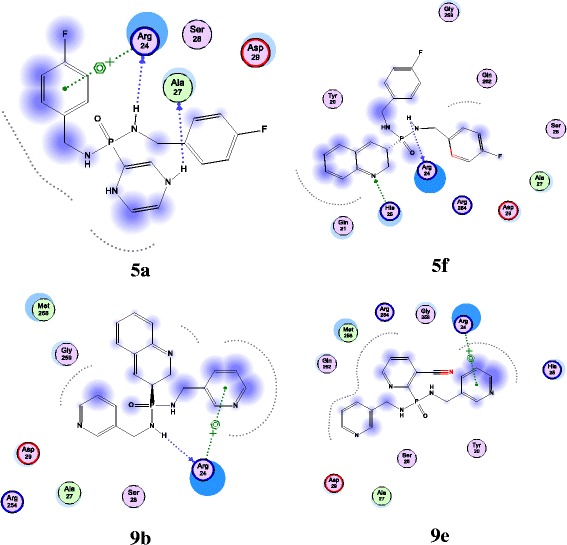
**Molecular docking complexes of 5a, 5f and 9b and 9e with PTP (PDB ID: 2F71)**

A total of 30 binding pose conformations were generated for each compound from docking simulations using MOE dock system. The free energy of binding of each ligand was ranked and assessed by London dG scoring function. The binding energies and hydrogen bond interactions of each Receptor-Ligand complexes were studied and the information is tabulated in Table 2. The best lowest docking score -11.810 Kcal/mol was observed for the compound **9e** and second the highest docking score of -9.813 Kcal/mol was observed for **9a**. The remaining compounds are also showing better docking scores indicating the good affinity levels between the receptor and compounds. The binding mode orientations of **9e** ligand-receptor complex are showing that the ligand is interacting with the binding site with the help of a single arene cat ionic interaction with Arg24 residue. Hydrogen bond interactions were not seen for **9e** in the complex. It was observed from all docking complexes that Arg24 residue is playing a major role in interacting with almost all of the compounds. In addition with **9e**, the arene cat ionic interaction was also observed with the compounds **5d**, **9b**, **9c**, **9d** and **9f**. More over in all of these complexes the arene cat ionic interaction was contributed by Arg24 residue only. This indicates that the aromatic rings of the compounds are highly influencing them to interact with the Arginine residue. The hydrogen bond interactions were not observed for **9d** and **9e** where as such bonds were observed in the remaining docking complexes (Figure 3 and Additional file 1: Figure S1-S12). However, **9d** and **9e** are also showing satisfactory docking scores along with remaining compounds. So, finally it can be predicted from these studies that all these compounds have the ability to bind with PTP-1B and inhibits its activity.

**Table 2:** **Molecular docking of the title compounds (5a-f and 9a-f) into the PTP biding domain**

**Ligand**	**Docking score (Kcal/mol)**	**No.H-bonds**	**Interacting residues**	**H-bond length (Å)**
**5a**	−10.5251	2	Arg 24	2.4
			Ala 27	2.8
		Arene cat ionic interaction	Arg 24	
**5b**	−11.3541	1	Gln262	3.2
**5c**	−10.2642	2	Arg24	3.2
			Arg254	3.0
**5d**	−10.1672	1	Arg24	2.6
		Arene cat ionic interaction	Arg24	
**5e**	−10.3299	2	Arg24	2.8
			Arg24	2.9
**5f**	−11.4417	1	Arg24	2.5
			His25	2.3
**9a**	−9.8136	1	Arg24	2.3
**9b**	−11.4253	1	Arg24	2.8
		Arene cat ionic interaction	Arg24	
**9c**	−10.1006	1	Arg24	2.7
		Arene cat ionic interaction	Arg24	
**9d**	−11.2717	Arene cat ionic interaction	Arg24	
**9e**	−11.8104	Arene cat ionic interaction	Arg24	
**9f**	−10.5172	1	Arg24	2.0
		Arene cat ionic interaction	Arg254	

Major strength of interaction is contributed by arene cat ionic interactions.

### ADMET results

**Figure 4:**
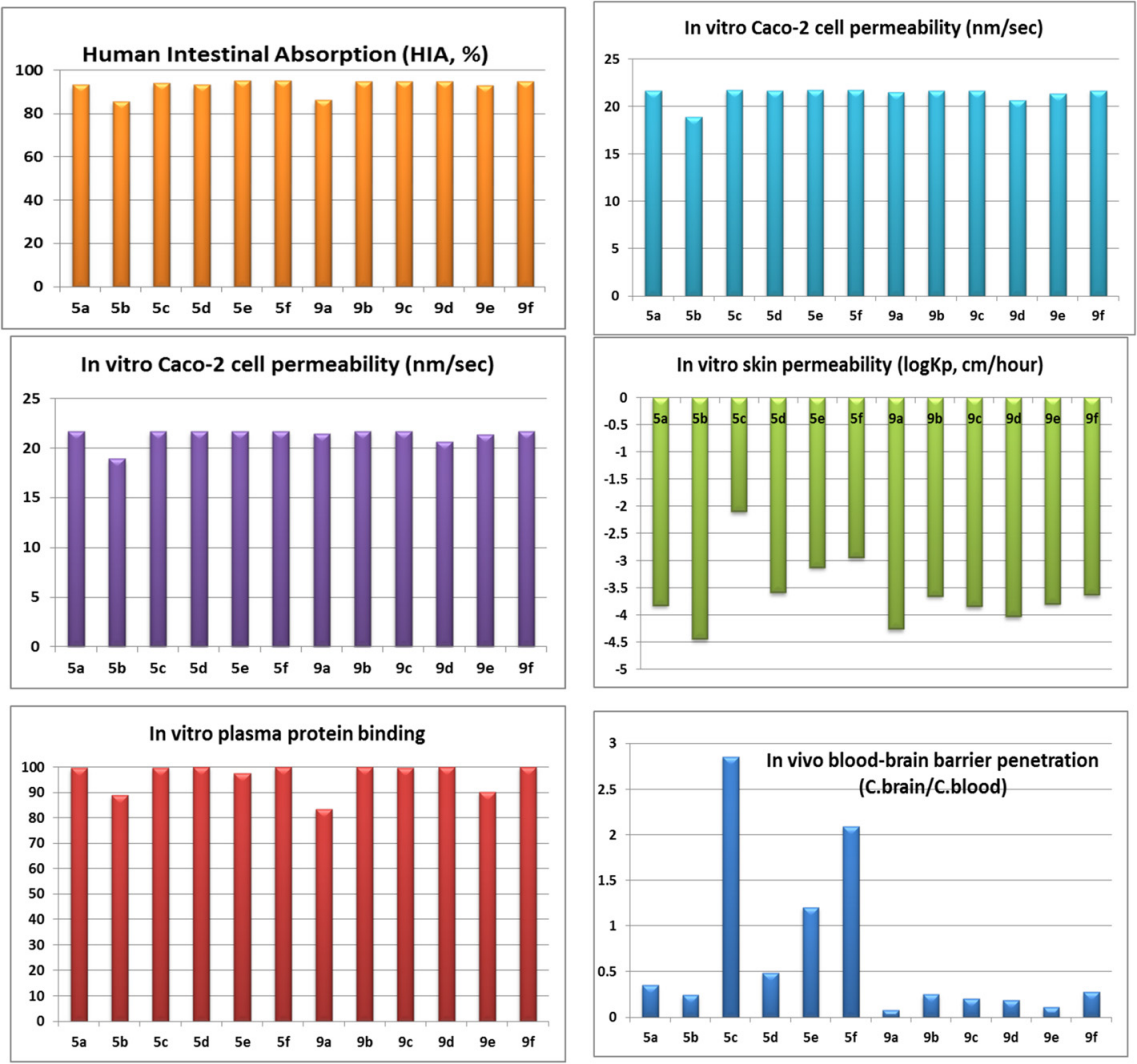
**ADMET results of title compounds 5a-f and 9a-f.**

Human intestinal absorption, Caco-2 cell permeability, MDCK cell permeability, BBB penetration, skin permeability and plasma protein binding abilities of the title compounds were calculated by PreADMET server and the results presented in Figure 4.

The HIA results demonstrate the best absorption of the title compounds **5a-f** and **9a-f** into Human Intestine. Weak plasma protein binding results represent their virtuous properties such as diffusion or transport across cell membranes, interaction with a pharmacological target and excretion. This is due to, generally the drugs less bound to plasma protein exist freely for diffusion or transport across cell membranes and also for interaction with a pharmacological target. The title compounds **9a-j,** altogether showed moderate cellular permeability against Caco-2 cells. The compound **5b** exhibited medium MDCK cellular permeability. In turn all the above parameters represent their good excretion, disposition and efficacy values in the human body.

The Blood-Brain Barrier (BBB) penetration is represented as BB = [Brain]/[Blood], where [Brain] and [Blood] are the steady-state concentration of radio labeled compound in brain and peripheral blood. Predicting BBB penetration helps to know whether the compounds able to pass across the blood-brain barrier or not. This parameter expresses the BBB penetration capacity and absorption rate of compound to CNS. All the compounds were observed to be having moderate absorption to CNS.

The skin permeability is a crucial parameter that can define the transdermal delivery of the compound as the risk assessment during accidental contact with the skin. The skin permeability values are defined as logKp, cm/hr for all the compounds, where Kp = Km*D/h. **Km** is distribution coefficient between stratum corneum and vehicle, **D** is average diffusion coefficient (cm^2^/h) and **h** is thickness of skin (cm) [41].

### Pharmacology

The title compounds were assessed for anti-oxidant and anti-hyperglycemic activity. The detailed discussion regarding the assessment method is demonstrated as follows.

### Antioxidant activity

#### Free radical 2,2-diphenyl-1-picrylhydrazyl (DPPH) scavenging activity

**Figure 5:**
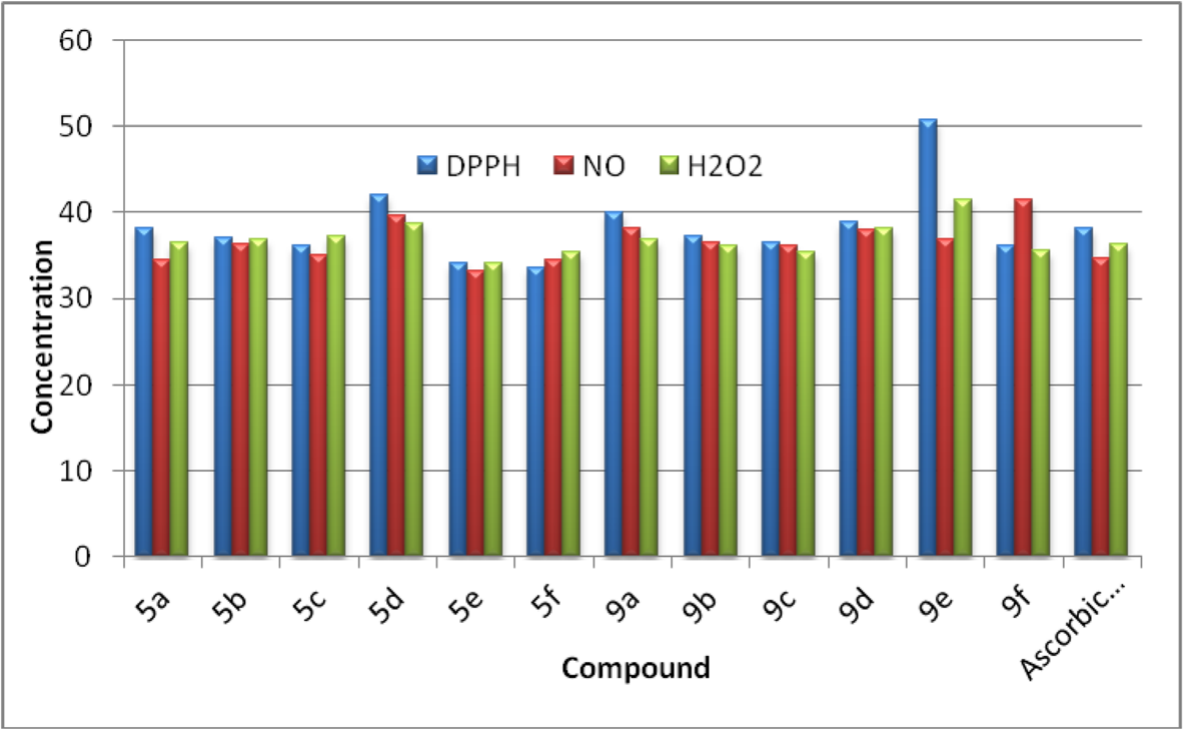
**IC**_**50**_ **values in μg/mL of the title compounds on DPPH scavenging activity, Nitric oxide radical scavenging activity and superoxide radical (O**_**2**_^**−**^ **) scavenging activity.**

DPPH is usually used as a reagent to evaluate free radical scavenging activity of antioxidants [42]. DPPH is a stable free radical and accepts an electron or hydrogen radical to become a stable diamagnetic molecule [43]. The reduction capability of DPPH radical is determined by the decrease in absorbance at 517 nm induced by antioxidants. In the present study, Ascorbic acid was used as a standard, the title compounds **5a-f** and **9a-f** were able to reduce the stable radical DPPH to the yellow-colored diphenylpicrylhydrazine. The compounds were evaluated at four different concentrations of 50 μg/mL, 75 μg/mL, 100 μg/mL and 150 μg/mL and the IC_50_ values were determined from these evaluations. The scavenging effect of title compounds as compared to standard with the DPPH radical is in the following order **5f** >**5e** >**9f** > Ascorbic acid >**5c** >**9c** and the remaining compounds showed less effect than these compounds. The compounds showed almost all same order at all concentrations and the complete results are given in Additional file 1. The IC_50_ value of each compound was considered as the concentration (μg/mL) of the compound at which 50% of DPPH reduction was observed. These results are presented in Figure 5 and in Additional file 1: Table S5.

#### Assay of Nitric oxide radicals scavenging activity

In the current investigation, newly synthesized compounds exhibited an excellent NO radicals scavenging activity. The compounds were evaluated at four different concentrations of 50 μg/mL, 75 μg/mL, 100 μg/mL and 150 μg/mL and the IC_50_ values were determined from these evaluations. Amongst the title compounds **5e** > BHT >**5f** >**5a** >**5c** >**9f** have exerted significant inhibitory activity and the remaining compounds exhibited less effect than these compounds on radicals that are generated *in vitro* and the complete results are given in Additional file 1. The IC_50_ value of each compound was considered as the concentration (μg/mL) of the compound at which 50% of NO reduction was observed. These results are presented in Figure 5 and in Additional file 1: Table S6.

#### Assay of superoxide radical (O_2_^−^) scavenging activity

Superoxide radical is known to be a very harmful species to cellular components as a precursor of more reactive oxygen species [44]. The superoxide radical is known to be produced *in vivo* and can result in the formation of H_2_O_2_*via* dismutation reaction. Moreover, the conversion of superoxide and H_2_O_2_ into more reactive species, for instance, the hydroxyl radical, has been thought to be one of the unfavorable effects caused by superoxide radicals [45]. The newly synthesized compounds are efficient scavengers for the superoxide radical generated in riboflavin–NBT–light system *in vitro* and their activity is in comparable to that of Ascorbic acid. The compounds were evaluated for their scavenging effects at four different concentrations of 50 μg/mL, 75 μg/mL, 100 μg/mL and 150 μg/mL and the IC_50_ values were determined from these evaluations. The scavenging effects of the compounds are in the following order **5e** >**5f** >**9f** >**9c** > Ascorbic acid and the remaining compounds exhibited less scavenging effect than these compounds on radicals that are generated *in vitro.* The compounds exhibited almost all same order at all concentrations and the complete results are presented in Additional file 1. This result clearly indicates that the tested compounds have a noticeable effect on scavenging superoxide radical. The IC_50_ value of each compound was considered as the concentration (μg/mL) of the compound at which 50% of NO reduction was observed. These results are presented in Figure 5 and in Additional file 1: Table S7.

In over view of observation, the compounds **5e, 5f, 9c, 9f** are showing the better antioxidant activity, it may be due to the presence of quinolone group in the structures of the title compounds. These results are supported by the previous reports of Shridhar et al. of the antioxidant activity of eight substituted quinolines [46].

### The proposed mechanism for the DPPH radical scavenging activity with the title compounds

**Figure 6:**
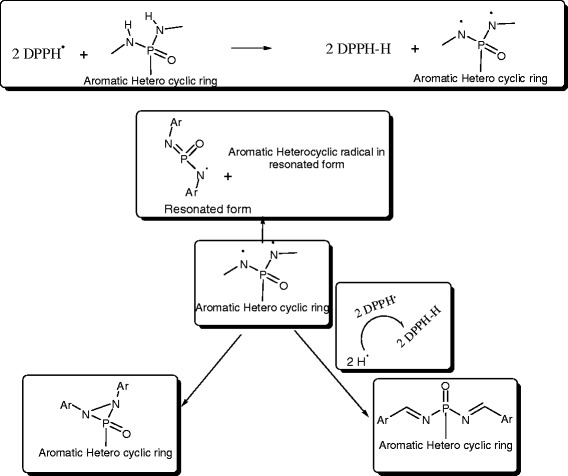
**The proposed mechanism for the DPPH radical scavenging activity with the title compounds [** 47 **].**

All the title compounds are containing atleast two N-H functional groups in their structure. These N-H groups are playng the main role in the abridging of free radicals, there by oxidative stress decreases. This mechanisim was supported by previous reports. This reduction of oxidative stress is the basis for mechanism for the antihyperglycemic actitivity (Figure 6).

### Anti-hyperglycemic activity

**Figure 7:**
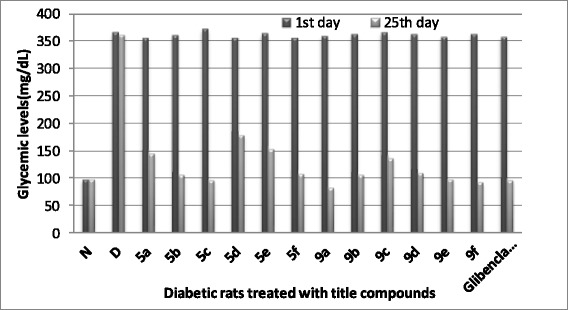
**Anti-diabetic activity of compounds 5a-f and 9a-f in STZ induced diabetic rats.**

All the title compounds showed significant anti-diabetic activity in the diabetes-induced rats when compared with the standard Glibenclamide. All the rats were kept in the observation for 25 days and seasoned the glycemic levels (mg/dL) for every four days after administration of drug. The diabetic rats showing glucose levels ≥350 mg/dL were taken for the experiment on the first day. On the fourth day, the glucose levels were almost all decreased i.e 221 ± 3.25 (**9a**) <242 ± 3.42 (**9f**) <244 ± 2.35 (**5f**) <245 ± 2.56 (Standard) <245 ± 4.86 (**9b**) <250 ± 3.24 (**5c**). On the eighth day the compounds **9a** (152 ± 3.46) and **9f** (195 ± 3.48) showed the least glycemic levels than the other compounds. On the 12^th^ day, the compound **9a** induced rats showed the normal glycemic levels (120 ± 3.15) and compound **9b** (146 ± 1.95)**, 9f** (153 ± 2.32)**, 5c** (165 ± 2.76)**,** Glibenclamide (166 ± 2.46) and **9e** (168 ± 2.68) induced rats gave moderate glycemic levels. On the 16^th^ day only the compound **9a** (103 ± 1.47) gave the good result. The remaining compounds gave the moderate glycemic levels except **5a, 5d, 5e** and **9c.** On the 20^th^ day all the compounds showed normal glycemic levels but **9d** gave moderate and **5a, 5d, 5e** and **9c** gave the high glycemic levels. Finally on the last day, all the title compounds gave excellent results with glycemic levels in between 82 ± 1.58 (**9a**) to 109 ± 1.23 (**9d**) apart from **5a, 5d, 5e** and **9c**. The detailed observation of results of all title compounds are reproduced in Table 3 (Figure 7).

**Table 3:** **Anti-diabetic activity of compounds 5a-f and 9a-f in STZ induced diabetic rats**

**Compound**	**Glycemic levels(mg/dL) at different time intervals after drug administration to mice**						
	**1** ^**st**^ **day**	**4day**	**8** ^**th**^ **day**	**12** ^**th**^ **day**	**16** ^**th**^ **day**	**20** ^**th**^ **day**	**25** ^**th**^ **day**
N	98 ± 1.42^**g**^	97 ± 1.42^**g**^	98 ± 1.42^**g**^	98 ± 1.42^**g**^	99 ± 1.42^**g**^	99 ± 1.42^**g**^	98 ± 1.42^**g**^
D	365 ± 3.22^**ng**^	364 ± 2.85^**ng**^	366 ± 4.54^**ng**^	360 ± 3.72^**ng**^	364 ± 2.18^**ng**^	362 ± 4.36^**ng**^	361 ± 1.68^**ng**^
**5a**	355 ± 2.55^**n**^	280 ± 4.85^**ng**^	225 ± 4.58^**ng**^	198 ± 4.25^**ng**^	172 ± 3.25^**ng**^	165 ± 3.85^**ng**^	145 ± 2.76^**ng**^
**5b**	360 ± 3.44^**n**^	270 ± 3.95^**ng**^	210 ± 4.34^**n**^	179 ± 3.25^**ng**^	135 ± 1.22^**n**^	110 ± 1.85^**n**^	105 ± 2.32^**n**^
**5c**	372 ± 4.22^**ng**^	250 ± 3.24^**n**^	208 ± 2.85^**n**^	165 ± 2.76^**n**^	138 ± 2.44^**n**^	116 ± 3.08^**n**^	95 ± 2.25
**5d**	356 ± 4.44^**n**^	312 ± 4.21^**ng**^	250 ± 3.59^**ng**^	221 ± 2.65^**ng**^	200 ± 3.26^**ng**^	193 ± 2.44^**ng**^	178 ± 1.42^**ng**^
**5e**	364 ± 3.88^**n**^	272 ± 2.66^**ng**^	236 ± 2.88^**ng**^	202 ± 3.11^**ng**^	182 ± 1.35^**ng**^	169 ± 2.52^**ng**^	152 ± 1.43^**ng**^
**5f**	355 ± 3.14^**n**^	244 ± 2.35^**n**^	212 ± 3.16^**n**^	173 ± 2.42^**ng**^	142 ± 2.35^**ng**^	125 ± 1.56^**n**^	108 ± 1.34^**n**^
**9a**	359 ± 2.37^**n**^	221 ± 3.25^**ng**^	152 ± 3.46^**ng**^	120 ± 3.15^**ng**^	103 ± 1.47^**g**^	95 ± 1.25^**g**^	82 ± 1.58^**ng**^
**9b**	362 ± 4.92^**n**^	245 ± 4.86^**n**^	205 ± 2.83^**n**^	146 ± 1.95^**ng**^	132 ± 2.2^**ng**^	123 ± 2.63^**n**^	105 ± 1.73
**9c**	365 ± 5.22^**ng**^	306 ± 3.58^**ng**^	295 ± 3.64^**ng**^	222 ± 2.95^**ng**^	193 ± 1.45^**ng**^	162 ± 1.73^**ng**^	135 ± 1.34^**ng**^
**9d**	362 ± 1.85^**n**^	274 ± 4.23^**ng**^	224 ± 2.62^**ng**^	183 ± 3.18^**ng**^	152 ± 1.36^**ng**^	132 ± 1.8^**ng**^	109 ± 1.23^**ng**^
**9e**	357 ± 2.33^**n**^	255 ± 3.82^**ng**^	205 ± 2.47^**n**^	168 ± 2.68^**n**^	132 ± 1.45^**ng**^	114 ± 1.37^**n**^	98 ± 1.3
**9f**	363 ± 3.09^**n**^	242 ± 3.42^**n**^	195 ± 3.48^**ng**^	153 ± 2.32^**ng**^	131 ± 1.76^**ng**^	108 ± 1.92^**ng**^	92 ± 1.54^**ng**^
G	357 ± 2.95^**n**^	245 ± 2.56^**n**^	210 ± 2.45^**n**^	166 ± 2.46^**n**^	137 ± 1.95^**n**^	118 ± 1.83^**n**^	95 ± 2.15^**n**^

with N = Normal rats untreated, D = Diabetic rats untreated G = Glibenclamide.

‘^**n**^’Represents significant difference on the respective day with normal rat group at p < 0.05.

‘^**g**^’Represents significant difference on the respective day with glibenclimade rat group at p < 0.05.

The data values were analyzed using one way ANOVA.

The molecules which were bound at Arg24 of Protein tyrosine phosphatase (PTP) gave the potential anti-hyperglycemic properties against Diabetic rats. From these results, compound **9a** can be stated as an effective anti-hyperglycemic compound among all as it exerted its effect in the earlier days among all. This may be due to the presence of two methoxy groups on the triazine moiety which are binding at Arg24 of PTP that can make it more reactive and effective. On the 20^th^ day **5b, 5c, 9a, 9e** and **9f** compounds gave normal glycemic levels, it may be due to the presence of structural moieties like uracil, trifluoromethyl, dimethoxytriazine, nicotinonitrile and quinoline moieties respectively. On the othe other hand the same ligand groups are binding with PTP at Arg24. But a few compounds exhibited moderate results though they contain the quinoline and isoquinoline structures. On an overall, all the compounds have shown good anti-diabetic activities by 25^th^ day except **5a, 5d, 5e** and **9c**. Over again, these results from molecular docking studies and *in vivo* assays of the title compounds supporting for previous reports that development of PTPs inhibitors is very ease for diabetes prevention [48].

## Conclusion

Lanthanum (III) chloride is stated as an efficient catalyst for the Michaelis-Arbuzov reaction and for the synthesis of the title compounds **5a-f** and **9a-f** by two-step reaction. The molecular descriptors of all the compounds from Lipinski parameters explained their drug likeliness and suggesting them as safer drugs. The descriptors also indicate that they are all not harmful to the host system because of their optimal logP values. The molecular docking study revealed their strong ability to interact with the target and inhibits its activity, there by predicting their anti-diabetic activity. PreADMET results demonstrated that the title compounds exhibit good absorption, permeability, penetration abilities in the human body. This prediction is confirmed by *in vivo* screening of these compounds in the diabetic induced rat models where the test compounds exhibited significant antihyperglycemic activity comparative to the standard Glibenclamide. Almost all the compounds brought the glycemic levels to normal on the 25^th^ day. Especially, **9a** was shown normal glycemic levels on 12^th^ day. On the 20^th^ day **5c, 5b, 9a, 9e** and **9f** were shown normal glycemic levels. All the compounds exhibited good diabetic levels on 25^th^ day except **5a, 5d, 5e** and **9c**.

### Supporting information

Supporting Tables of the antioxidant activity and supporting Figures are given in the supporting information as Additional file 1.

## Additional file

Additional file 1:
**Construction of 3D-models and ligand database of the compounds.** Lipinski Descriptors. **Table S5.** DPPH radical scavenging activity of the compounds **5a-f** and **9a-f. Table S6.** Nitric oxide (NO)scavenging activity of the compounds **5a-f** and **9a-f. Table S7.** Hydrogen peroxide (H_2_O_2_) scavenging activity of the compounds **5a-f** and **9a-f. Figure S1:** molecuar docking complex of **5a. Figure S2:** molecuar docking complex of **5b. Figure S3:** molecuar docking complex of **5c. Figure S4:** molecuar docking complex of **5d. Figure S5:** molecuar docking complex of **5e. Figure S6:** molecuar docking complex of **5f. Figure S7:** molecuar docking complex of **9a. Figure S8:** molecuar docking complex of **9b. Figure S9:** molecuar docking complex of **9c. Figure S10:** molecuar docking complex of **9d. Figure S11:** molecuar docking complex of **9e. Figure S12:** molecuar docking complex of **9f**.
