# Supplementary material for: Novel heteroaryl phosphonicdiamides PTPs inhibitors as anti-hyperglycemic agents
Source: Daru. 2014 Dec 27;22(1):76. doi: 10.1186/s40199-014-0076-3 (PMC4305230; doi:10.1186/s40199-014-0076-3)
Supplement: Additional file 1: — Construction of 3D-models and ligand database of the compounds. Lipinski Descriptors. Table S5. DPPH radical scavenging activity of the compounds 5a-f and 9a-f. Table S6. Nitric oxide (NO)scavenging activity of the compounds 5a-f and 9a-f. Table S7. Hydrogen peroxide (H2O2) scavenging activity of the compounds 5a-f and 9a-f. Figure S1: molecuar docking complex of 5a. Figure S2: molecuar docking complex of 5b. Figure S3: molecuar docking complex of 5c. Figure S4: molecuar docking complex of 5d. Figure S5: molecuar docking complex of 5e. Figure S6: molecuar docking complex of 5f. Figure S7: molecuar docking complex of 9a. Figure S8: molecuar docking complex of 9b. Figure S9: molecuar docking complex of 9c. Figure S10: molecuar docking complex of 9d. Figure S11: molecuar docking complex of 9e. Figure S12: molecuar docking complex of 9f. [file 40199_2014_76_MOESM1_ESM.docx]

**Additional file 1**

***Construction of 3D-models and ligand database of the compounds***

The three dimensional structures of all the compounds were constructed individually in MOE working environment and optimized under MMFF94x (Merck Molecular Force Field) force field enabling the potential energy terms for all bonded, Vander Waals and electrostatic interactions. Generalized Born implicit salvation model was enabled at a gradient of 0.05 and dynamics simulations were carried out applying Nose Poincare Anderson equational algorithm. Initial temperature was set to 30K and increased to a run time temperature of 300K. The stabilized conformations of the compounds obtained at the end of the simulations were used to construct a small ligand data base.

***Lipinski Descriptors***

The purpose of Lipinski descriptors is to calculate their properties that serve as numerical descriptions. The Lipinski suite of applications MOE is used to derive and evaluate the descriptors of the molecules that are stored in the database. Any of the molecular property can be considered as molecular descriptor and in turn they can be used to define the drug likeliness of the molecule. The descriptors Molecular weight, Hydrogen bond donors and Acceptors, LogP, Molar Refractivity, Surface area, Volume, Hydration energy, Polarizability, Gradient energy and Total energy were calculated using the QSAR-Descriptor module of MOE.

**Table S5.** DPPH radical scavenging activity of the compounds **5a-f** and **9a-f**

| Compound | % of activity in different concentration in µg/mL | | | | IC_50_ |
| --- | --- | --- | --- | --- | --- |
|  | 50 µg/mL | 75 µg/mL | 100 µg/mL | 150 µg/mL |  |
| **5a** | 65.55±0.56 | 73.18±0.58 | 76.53±0.65 | 81.14±0.65 | 38.13±0.64 |
| **5b** | 67.34±0.72 | 75.55±0.76 | 78.75±0.54 | 81.33±0.78 | 37.12±0.86 |
| **5c** | 69.16±0.68 | 77.48±0.72 | 79.89±0.75 | 85.78±0.86 | 36.14±0.76 |
| **5d** | 59.48±0.54 | 68.73±0.56 | 70.84±0.62 | 74.24±0.76 | 42.03±0.78 |
| **5e** | 73.32±0.83 | 80.62±0.86 | 83.35±0.86 | 88.86±0.94 | 34.09±0.92 |
| **5f** | 74.44±0.57 | 82.66±0.56 | 85.33±0.67 | 89.93±0.68 | 33.58±0.64 |
| **9a** | 62.44±0.95 | 70.32±0.88 | 72.55±0.74 | 78.72±0.84 | 40.03±0.76 |
| **9b** | 67.15±0.68 | 75.16±0.56 | 76.95±0.86 | 81.42±0.92 | 37.23±0.68 |
| **9c** | 68.53±0.85 | 76.22±0.76 | 78.38±0.58 | 81.45±0.68 | 36.48±0.86 |
| **9d** | 64.24±0.74 | 72.11±0.84 | 74.85±0.87 | 79.25±0.76 | 38.91±0.76 |
| **9e** | 49.34±0.67 | 58.45±0.76 | 60.74±0.94 | 65.14±0.86 | 50.66±0.68 |
| **9f** | 69.32±0.78 | 78.95±0.65 | 82.17±0.68 | 84.88±0.78 | 36.06±0.84 |
| Ascorbic acid | 65.48±0.96 | 78.16±0.97 | 81.54±0.84 | 85.16±0.96 | 38.17±0.95 |

**Table S6.** Nitric oxide (NO)scavenging activity of the compounds **5a-f** and **9a-f**

| Compound | % of activity in different concentration in µg/ mL | | | | IC_50_ |
| --- | --- | --- | --- | --- | --- |
|  | 50 µg/mL | 75 µg/mL | 100 µg/mL | 150 µg/mL |  |
| **5a** | 72.42±0.59 | 79.48±0.65 | 83.78±0.68 | 85.72±0.76 | 34.52±0.84 |
| **5b** | 68.82±0.68 | 76.58±0.94 | 78.47±0.76 | 80.46±0.84 | 36.32±0.67 |
| **5c** | 71.38±0.76 | 78.82±0.87 | 81.28±0.86 | 84.53±0.68 | 35.02±0.56 |
| **5d** | 63.14±0.56 | 70.86±0.98 | 74.38±0.94 | 77.72±0.92 | 39.59±0.68 |
| **5e** | 75.46±0.96 | 82.79±0.74 | 86.86±0.56 | 89.44±0.74 | 33.13±0.84 |
| **5f** | 72.44±0.78 | 80.72±0.56 | 84.44±0.84 | 86.44±0.64 | 34.51±0.74 |
| **9a** | 65.54±0.64 | 73.19±0.78 | 75.74±0.78 | 77.26±0.58 | 38.14±0.66 |
| **9b** | 68.48±0.58 | 75.73±0.68 | 79.48±0.98 | 82.48±0.86 | 36.50±0.84 |
| **9c** | 69.28±0.89 | 76.44±0.86 | 78.95±0.67 | 80.95±0.67 | 36.08±0.78 |
| **9d** | 65.77±0.84 | 73.23±0.94 | 75.92±0.76 | 78.85±0.94 | 38.01±0.94 |
| **9e** | 67.75±0.94 | 75.23±0.76 | 80.26±0.84 | 83.46±0.56 | 36.90±0.64 |
| **9f** | 60.35±0.56 | 79.14±0.69 | 82.86±0.86 | 86.75±0.76 | 41.42±0.78 |
| BHT | 72.24±0.74 | 82.45±0.86 | 85.95±0.74 | 88.78±0.84 | 34.60±0.86 |

**Table S7.** Hydrogen peroxide (H_2_O_2_) scavenging activity of the compounds **5a-f** and **9a-f**

| Compound | % of activity in different concentration in µg/ mL | | | | IC_50_ |
| --- | --- | --- | --- | --- | --- |
|  | 50 µg/mL | 75 µg/mL | 100 µg/mL | 150 µg/mL |  |
| **5a** | 68.47±0.68 | 76.55±0.72 | 80.82±0.68 | 85.34±0.56 | 36.51±0.87 |
| **5b** | 67.92±o.74 | 74.24±0.68 | 76.95±0.74 | 79.55±0.75 | 36.80±0.77 |
| **5c** | 67.23±0.84 | 75.35±0.84 | 77.84±0.86 | 80.19±0.88 | 37.18±0.66 |
| **5d** | 64.49±0.92 | 73.17±0.64 | 78.83±0.92 | 83.77±0.67 | 38.76±0.56 |
| **5e** | 73.42±0.56 | 80.56±0.56 | 83.92±0.56 | 89.28±0.87 | 34.05±0.56 |
| **5f** | 70.49±0.84 | 79.45±0.76 | 83.25±0.74 | 89.32±0.98 | 35.46±0.68 |
| **9a** | 67.95±0.65 | 75.84±0.86 | 80.58±0.68 | 86.65±0.56 | 36.79±0.92 |
| **9b** | 69.15±0.75 | 77.79±0.74 | 80.18±0.92 | 85.75±0.86 | 36.15±0.84 |
| **9c** | 70.66±0.88 | 78.08±0.96 | 81.78±0.64 | 87.83±0.68 | 35.33±0.54 |
| **9d** | 65.42±0.67 | 74.21±0.68 | 77.48±0.75 | 79.54±0.75 | 38.21±0.74 |
| **9e** | 60.24±0.86 | 68.69±0.67 | 70.33±0.67 | 72.15±0.68 | 41.50±0.84 |
| **9f** | 70.32±0.92 | 80.78±0.86 | 82.98±0.54 | 87.21±0.88 | 35.55±0.92 |
| Ascorbic acid | 68.84±0.64 | 76.89±0.98 | 81.26±0.92 | 85.14±0.67 | 36.33±0.67 |

**Figure S1**: molecuar docking complex of **5a**

**Figure S2:** molecuar docking complex of **5b**

**Figure S3:** molecuar docking complex of **5c**

**Figure S4:** molecuar docking complex of **5d**

**Figure S5:** molecuar docking complex of **5e**

**Figure S6:** molecuar docking complex of **5f**

**Figure S7:** molecuar docking complex of **9a**

**Figure S8:** molecuar docking complex of **9b**

**Figure S9:** molecuar docking complex of **9c**

**Figure S10:** molecuar docking complex of **9d**

**Figure S11:** molecuar docking complex of **9e**

**Figure S12:** molecuar docking complex of **9f**
